# Nutritional Composition, Total Phenolic Content, Antioxidant and α-Amylase Inhibitory Activities of Different Fractions of Selected Wild Edible Plants

**DOI:** 10.3390/antiox8070203

**Published:** 2019-07-01

**Authors:** Ziaul Hasan Rana, Mohammad Khairul Alam, Mohammad Akhtaruzzaman

**Affiliations:** 1 Department of Nutritional Sciences, Texas Tech University, Lubbock, TX 79409, USA; 2 Institute of Nutrition and Food Science, University of Dhaka, Dhaka-1000, Bangladesh

**Keywords:** antioxidants, α-amylase, Bangladesh, nutritional profile, total phenolic content, wild plants

## Abstract

Wild plants are considered the richest source of essential nutrients and other beneficial phytochemicals. Hence, the objective of this study was to evaluate the nutritional composition, antioxidant- and α-amylase inhibition activities of leaves and roots of selected Bangladeshi wild plants. These wild plants were found to have high fiber (13.78–22.26 g/100 g), protein (7.08–21.56 g/100 g) and ash (8.21–21.43 g/100 g) contents. The total phenolic and total flavonoid contents were significantly higher in the leaves than the roots. Additionally, antioxidant activity was evaluated using ferric-reducing antioxidant power, 2, 2-diphenyl-1-picrylhydrazyl radical (DPPH) and trolox equivalent antioxidant capacity assays and was strongly correlated with phenolic compounds. The leaf extracts of the selected plants also exhibited potent α-amylase inhibition (~71%) and were significantly higher than their root counterparts. Thus, the study findings concluded that the investigated plants were good sources of fiber, protein, mineral, natural antioxidant compounds and α-amylase inhibitors, and their increased intake could provide health benefits. The principal component analysis (PCA) of analyzed variables divided the samples into three clear groups, and the first two principal components accounted for 86.05% of the total data set variance.

## 1. Introduction

Vegetables are an integral part of the daily human diet and provide essential nutrients (vitamins and minerals) required for active and healthy life. They are a locally available and cheap source of nutrient-dense foods and considerably contribute to human health, nutrition and food security. The edible parts of vegetable plants include the leaves, roots, stems, fruits or seeds, and they can be consumed cooked as well as in raw forms. Evidence from epidemiological studies indicate that daily consumption of fruits and vegetables is correlated with a lower prevalence of many chronic diseases, including diabetes, infections, cardiovascular and neurological disorders and cancers [1,2,3]. Wild vegetables are considered to be a potential source of essential nutrients such as vitamin C, minerals, vitamins, proteins, fibers [4,5,6] and are also good dietary sources of antioxidants such as flavonoids and other polyphenolic constituents [4,7]. 

A diet low in antioxidants and high in processed foods (e.g., red meat) can augment the production of endogenous reactive oxygen species (ROS) [8,9,10] which can lead to many of the chronic diseases stated above [11]. This demonstrates the need for natural antioxidant compounds which can prevent the overproduction of ROS. It has been shown that there is an inverse relationship between morbidity and mortality from degenerative disorders and the ingestion of natural antioxidants [12]. Currently, wild or traditional plants, as a source of natural antioxidants, have received increased attention because of their ability to scavenge ROS and also as sources of trace elements, and their additional health properties, such as antidiabetic, antibacterial and anticancer activity, make them valuable for incorporation into the daily diet [13,14,15,16].

Phenolic compound-rich food consumption has also been shown to be inversely associated with type-2 diabetes [17]. By binding to the non-specific site of the enzyme, phenolic compounds can inactivate the starch-digesting enzyme α-amylase [18]. Thus, wild edible plants, as a source of high phenolic compounds, can inhibit α-amylase activity which subsequently decreases postprandial rises in blood glucose by suppressing the rate of glucose release and absorption in the small intestine [19].

Several wild edible plants are traditionally consumed along with staple foods, especially in rural areas and a few urban communities, in Bangladesh. These plants play a vital role in fulfilling the demand for nutritional, minerals and antioxidant compounds in the diet of indigenous communities [4,20]; besides these factors, they are also used in treating certain medical conditions, for example, diabetes, in these local tribes [21]. However, there is lack of information about the nutritional composition of wild edible plants in Bangladesh and their ability to inhibit digestive enzymes. Therefore, the aim of this study was to assess the proximate and mineral composition, antioxidant potential, and α-amylase inhibition activity of the leaves and roots of three indigenous wild edible plants (*Achyranthes aspera* L., *Eclipta alba* L., and *Vitex negundo* L.) consumed by different local communities in Bangladesh. The findings of the present study will provide the preliminary data on the nutritional and neutraceutical potential of wild edible plants in Bangladesh and thus could be incorporated into food composition databases and used for further utilization as dietary supplements and/or functional foods.

## 2. Materials and Methods

### 2.1. Reagents

Analytical-grade acetone, petroleum ether, n-hexane, dichloromethane, sodium carbonate, Folin–Ciocalteu reagent and acetic acid were purchased from Merck (Darmstadt, Germany). Gallic acid (Pub Chem CID:370) was purchased from Tokyo Chemical Industry Co., (Tokyo, Japan) and 2,20-azinobis (3-ethylbenothiazoline-6-sulfonic acid) diammonium salt (ABTS) was purchased from Wako Pure Chemical Industries, Ltd. (Osaka, Japan). *α*-amylase, 2, 2-diphenyl-1-picrylhydrazyl radical (DPPH), Tri(2-pyridyl)-s-triazine (TPTZ), trolox, potassium persulfate, and mineral standards were obtained from Sigma Aldrich (Steinheim, Germany). All chemicals used for the analysis were of analytical grade.

### 2.2. Sample Collection and Preparation

**Figure 1:**
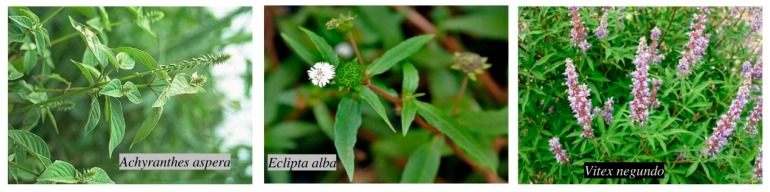
Photograph of selected samples.

To determine the proximate and mineral composition, total phenolic contents (TPC), total flavonoid contents (TFC), antioxidant capacities (DPPH, ferric-reducing antioxidant power (FRAP) and trolox equivalent antioxidant capacity (TEAC)), and α-amylase inhibition activity, three wild plant samples were collected from different locations in Bangladesh. Two to three samples (300–600 g) were collected for each of the wild plants from every growing location. These were then mixed to make three analytes or composite test samples. The study samples were *Achyranthes aspera* L. (Upat Lengra), *Eclipta alba* L. (Kalokeshi), and *Vitex negundo* L. (Nirgundi). The samples were selected based on their traditional use, by interviewing local people, in treating diabetes. The identification of the samples was confirmed by a taxonomist of the Department of Botany, University of Dhaka, who accompanied the collection team, after examining the morphological characteristics. Photographs of these samples are shown in Figure 1. After collection, the leaves and roots of the samples were separated and gently washed with tap water immediately to remove sand and other extraneous material before being washed with distilled water and then air-dried. Then, the samples were cut into small pieces and freeze-dried (il Shin lab.Co. Ltd., Korea). The freeze-dried samples were ground and homogenized into a fine powder using a grinder. The homogenized samples were sieved to obtain an even particle size, then placed in an air-tight zipper bag and stored at −20 °C until further analysis. 

### 2.3. Determination of Proximate Composition

The proximate composition (moisture, total protein, total fat, total dietary fiber including soluble and insoluble, ash and total available carbohydrate content) of the selected samples was estimated according to the method described previously [22]. Moisture and ash contents of the sample were calculated by the weight difference method, whereas the total fat content of the samples was estimated by the Association of Official Analytical Chemists (AOAC) method using petroleum ether as solvent. The total protein content was determined by using the micro-Kjeldhal method (nitrogen content of the samples × 6.25). The gravimetric method was utilized for the estimation of total dietary fiber (soluble and insoluble). Total available carbohydrate contents were calculated by difference using the formula below:

Carbohydrate content (%) = 100 − [total protein (%) + ash content (%) + total fat (%) + total fiber (%)].(1)

### 2.4. Determination of Mineral Composition

Mineral concentrations in the plants sample were calculated by using an atomic absorption spectrophotometric method described previously [23]. Briefly, approximately 500 mg of plant samples after drying were subjected to wet digestion with nitric acid and perchloric acid (2:1 ratio) in an auto-digestor at 325 °C to accelerate the discharge of mineral in the plant matrix. After digestion and appropriate dilution, the digested sample was aspirated into an air–acetylene flame to burn the elements into atomic components, which were then detected in a spectrophotometer at their relevant wavelengths. Proportions of calcium, magnesium, sodium, zinc, copper and iron were evaluated by atomic absorption spectrophotometry (Model-AA-7000S, Shimadzu, Tokyo, Japan). The amount of potassium was determined by flame photometry (Jenway flame photometer model PFP7, Origin UK). A standard calibration curve was plotted for each of the minerals using the respective mineral standard obtained from Sigma Chemical Co., USA. 

### 2.5. Plant Extraction

The extraction of plants was carried out according to the previously described procedure using methanol and 1N HCl [24], and the extract was stored at 4 °C for the determination of TPC, TFC, antioxidant activity and α-amylase activity.

### 2.6. Determination of Total Phenolic Content

The TPC in plant extracts was estimated by the Folin–Ciocalteu colorimetric method as described previously [22,24]. Briefly, for each sample, 150 μL of plant extracts were taken in test tubes. To this, 900 μL distilled water was added. 225 μL of diluted Folin–Ciocalteu reagent (2-fold) was added to the solution and allowed to stand for 5 min at room temperature. Then, 1.125 mL of 2% Na_2_CO_3_ solution was added, mixed well and left for 15 min at room temperature. Finally, the absorbance was measured at 750 nm by a UV-VIS spectrophotometer (UV-1800, Shimadzu, Kyoto, Japan). The TPC was calculated using a standard curve based on gallic acid. Results were expressed as milligrams of gallic acid equivalent (GAE) per gram dry weight (DW) (mg GAE/g DW). 

### 2.7. Determination of Total Flavonoid Content

The TFC was estimated by means of the colorimetric method according to Miao et al. [25] with slight modification. Briefly, 250 µL of the extract was mixed with 1.125 ml of distilled water in a test tube. To these, 75 µL of 5% NaNO_2_ solution was added. After 6 min, 150 µL of 10% AlCl_3_·6H_2_O solution was added. The solution was left to stand for another 5 min, and 500 µL of 1 M NaOH was added. Finally, the mixture was vortexed, and the absorbance was measured immediately at 510 nm by a UV-VIS spectrophotometer (UV-1800, Shimadzu, Kyoto, Japan). The TFC in the plant extract was calculated using a standard curve based on quercetin and results were expressed as milligrams quercetin equivalent (QE) per gram of dry weight (mg QE/g DW).

### 2.8. Evaluation of Antioxidant Capacities

#### 2.8.1. DPPH Free Radical Scavenging Assay

The antioxidant activity of the plant extracts was evaluated by utilizing 2,2-diphenyl-1-pycrylhydrazyl (DPPH) free radical according to Alam et al. [24]. The DPPH free radical inhibition capacity was calculated according to the following equation:

% DPPH inhibition = ((1 − ((Abs_sample_ − Abs_blank_)/(Abs_control_ − Abs_blank_))) × 100 (2)

where Abs_blank_ is the absorbance of the blank (containing only methanol), Abs_control_ is the absorbance of the control reaction (containing all reagents minus plant extracts), and Abs_sample_ is the absorbance of the plant extracts. The plant extract concentration required for the 50% inhibition of DPPH free radical (IC_50_) was estimated from the dose–response graph plotted with percentage inhibition and concentrations of plant extract.

#### 2.8.2. Ferric Reducing Antioxidant Power (FRAP) Assay

This assay was carried out according to Miao et al. [25] with little modification. Briefly, the FRAP reagent was made from by combining 10 mmol/L 2,4,6-tripyridyls-triazine (TPTZ) solution, 300 mmol/L acetate buffer (pH 3.6), and 20 mmol/L FeCl_3_ solution in a ratio of 1:10:1 (*v/v*), respectively. The FRAP reagent was freshly prepared and was incubated at 37 °C in a water bath before using. 100 µl of plant extracts were added to 3 mL of the FRAP reagent. The mixture was vortexed, and absorbance of the solution was then measured at 593 nm (UV-1800, Shimadzu, Kyoto, Japan) after incubating at 37 °C for 30 min. Various concentrations (50–600 µmol/L) of Fe^2+^ solution was used to prepare the standard curve. The results were expressed as µmol Fe^2+^ per gram of dry weight (µmol Fe^2+^/g DW).

#### 2.8.3. Trolox Equivalent Antioxidant Capacity (TEAC) Assay

This assay was performed by the advanced ABTS^•+^ method as described by Miao et al. [25] with little modification. ABTS^•+^ radical cation was produced by dissolving ABTS and potassium persulfate in distilled water to give a final concentration of 7 mmol/L and 2.45 mmol/L, respectively. The solutions were mixed, and the reaction mixture was left in the dark at room temperature for 24 h. The ABTS^•+^ solution was diluted with distilled water to an absorbance of 1.00 ± 0.03 at 734 nm. Then, 100 µL of plant extracts were added to 3.8 mL of diluted ABTS^•+^ solution and the solutions were kept in the dark for 10 min. After 10 min, the absorbance was read at 734 nm by a UV-VIS spectrophotometer (UV-1800, Shimadzu, Kyoto, Japan) against the blank (distilled water). The trolox solution of various concentrations (0–15 µmol/L) was used to prepare the standard curve, and the results were expressed as µmol trolox per gram of dry weight extract (µmol trolox/g DW).

### 2.9. α-Amylase Inhibitory Assay

The α-amylase inhibitory activity of the plant samples was performed by the modified starch iodine method described by Hossain et al. [26]. Briefly, 100 µL of plant extracts (0.25 mg/mL) were taken in test tubes. To each test tube, an aliquot of 2 μL of α-amylase was added and incubated for 10 min at 37 °C. After incubating, 1% starch solution (20 μL) was added. Then, the mixture was incubated again for 60 min at 37 °C. After that, 20 μL of 1% iodine solution was added to the mixture. The absorbance of the mixture was taken at 565 nm, after the addition of 1 mL distilled water. Acarbose, a known α-amylase inhibitor, was used as a standard. The α-amylase inhibitory activity was calculated and expressed as percentage inhibition using the following formula:

(%) α-amylase Inhibition = (1 − (Abs_sample_/Abs_control_)) × 100 (3)

where Abs_control_ is the absorbance of the control reaction (containing all reagents minus plant extracts or acarbose) and Abs_sample_ is the absorbance of the plant extracts or acarbose.

### 2.10. Statistical Analysis

All experiments were carried out in three replicates and presented as mean ± standard deviation (SD) using Minitab version 18.0. (Minitab Inc., State College, PA, USA). One-way analysis of variance (ANOVA) and principal component analysis (PCA) were performed to check the differences between the nutrient contents, TPC, TFC, antioxidant activity, and α-amylase inhibition activity among the plant samples. The differences were declared significant at a level of *p* < 0.05. The Dunnett test to compare with control for α-amylase activity and Pearson correlation among variables were also calculated.

## 3. Results and Discussion

### 3.1. Proximate Composition

Table 1 summarizes the proximate composition, macro mineral (Ca, Na, K Mg) and micro mineral (Fe, Zn, Cu) content in the leaves and roots of the investigated wild plants. 

The moisture content of the wild plants ranged from 82.78 ± 2.68 to 88.13 ± 1.55 g/100g fresh weight (FW) in the leaves and 55.44 ± 2.22 to 70.41 ± 2.11 g/100g FW in roots, which is in accordance with results reported by other authors [4,5]. On the other hand, Satter et al. [20] reported a higher content of moisture in some other wild plants of Bangladesh.

Protein contents were found between 18.13 ± 1.67 to 21.56 ± 1.10 g/100g DW in leaves and 7.08 ± 0.33 to 13.21 ± 0.93 g/100 g DW in roots, which is opposite to the findings of other studies [4,27]. However, our findings are similar to results observed by Satter et al. [20] and higher than those reported by Gupta et al. [5].

The contents of fat in this study were 1.88 ± 0.20 to 3.13 ± 0.51 g/100 g DW and 0.89 ± 0.07 to 1.12 ± 0.10 g/100 g DW in leaves and roots, respectively. These values are less than those reported by other authors [4,20,27]. 

The ash contents in the leaves and roots were observed from 19.78 ± 0.42 to 21.43 ± 0.33 g/100 g DW and 8.21 ± 0.61 to 17.37 ± 0.51 g/100 g DW, respectively. Afolayan & Jimoh [4] also reported similar values, whereas Gupta et al. [5] and Satter et al. [20] found lower contents than ours.

Dietary fiber content was in the range of 18.65 ± 1.23 to 20.28 ± 0.92 g/100 g DW in leaves and 13.78 ± 1.34 to 22.26 ± 0.56 g/100 g DW in roots, which is higher than that reported by previous studies [4,20]. Regional or other factors could be an explanation for these variations in proximate composition, as indicated elsewhere [22].

Amounts of total available carbohydrate (CHO) were documented to be 36.21 ± 0.63 to 39.91 ± 1.85 g/100 g DW in leaves and 51.92 ± 1.50 to 65.54 ± 1.78 g/100 g DW in roots. Satter et al. [20] found higher values of total CHO in the leaves of other wild plants of Bangladesh, whereas Afolayan & Jimoh [4] and Seal [27] stated lower values than ours in South African and Indian wild edible plants. Thus, the proximate composition of wild plants grown in Bangladesh contain similar or even higher contents of specific nutrients to those of other wild plants growing in different global areas. These wild plants, as a source of high fiber, protein and ash, and with a low fat content, could be incorporated into weight control diet for obese people.

### 3.2. Mineral Composition

The mineral (macro and micro) composition of the studied wild plants is presented in Table 1, and the results revealed that these wild plants were rich in a wide variety of minerals including Na, K, Mg, Ca, Fe, Zn, and Cu. *Vitex negundo* leaf was found to contain higher amounts of Na and Fe, whereas Mg and Zn contents were higher in the leaf of *Achyranthes aspera*. *Eclipta alba* leaf was found to be a better source of potassium (5174.82 ± 5.74 mg/100 g DW), calcium (2221.33 ± 6.83 mg/100 g DW) and Cu (2.33 ± 0.07 mg/100 g DW) than others (Table 1). The roots of the plant had a relatively lower amount of minerals than their corresponding leaves (*p* < 0.0001). Mg and Cu values in the plant samples were found to be comparable to the values reported by several authors [4,20]. However, the concentrations of other minerals were much higher than those reported for other wild plant varieties [20,27], and some values were less than those stated by Afolayan & Jimoh [4]. These wild plants contain comparable or higher amounts of minerals than those documented in some commonly consumed vegetables such as spinach, cauliflower, cabbage, and lettuce and other cultivated vegetables [28]. Thus, the selected wild plants could potentially be utilized as a good source of major and trace minerals required for normal body function and maintenance.

### 3.3. Total Phenolic and Flavonoid Contents

The TPC of the leaf and root extracts of the studied plants was determined by the Folin–Ciocalteu method. The plant samples undertaken in this study showed the presence of TPC in ranges of 2.46 ± 0.06 to 8.45 ± 0.15 mg GAE/g DW, and 55.32 ± 0.47 to 72.11 ± 0.73 mg GAE/g DW for the roots and leaves, respectively (Table 2). The leaves of the samples were higher in TPC than their roots (*p* < 0.0001). The leaf and root of *Vitex negundo* showed the highest (72.11 ± 0.73 mg GAE/g DW) and lowest (2.46 ± 0.06 mg GAE/g DW) content of TPC, respectively. 

The TFC of the root and leaf extracts of the samples was estimated by flavonoid–aluminum chloride conjugation method and is presented in Table 2. The estimation of TFC in different samples revealed values from 1.22 ± 0.09 to 4.88 ± 0.31 mg QE/g DW, and 31.55 ± 0.25 to 80.23 ± 0.55 mg QE/g DW for the roots and leaves, respectively (Table 2). The highest content (80.23 ± 0.55 mg QE/g DW) was observed in *Achyranthes aspera* leaf extract, while *Vitex negundo* root extract exhibited the lowest (1.22 ± 0.09 mg QE/g DW). Like TPC, TFC was also higher in leaves than roots (*p* < 0.0001). However, some studies reported higher contents of TPC and TFC in stems and roots than leaves, which is contradictory to our observation [29].

To our knowledge, there are little or no available data in the literature about the TPC and TFC of the selected plants; thus, only a few papers could be found related to the TPC and TFC of other species of the same families. The leaf extract of *Vitex negundo* has recently been reported to contain 89.71 mg GAE/g of TPC and 63.11 mg QE/g of TFC [30], which are relatively higher values than our study. Shahat et al. [31] reported TPC in the range from 11 to 56 mg GAE/g of DW for different species of Asteraceae family, which is a lower value than our findings. Compared to a study by Nana et al. [32], using *Amaranthus cruentus* and *Amaranthus hybridus* of the Amaranthaceae family, we found lower TPC and TFC values in our sample of the Amaranthaceae family. We also found higher TPC in our study than some frequently consumed local vegetables [33]. On the other hand, our observed content was lower than some reported Asian vegetables [7,34]. However, it is well recognized that several factors, such as species, plant tissue, temperature, water stress and light conditions, as well as phenological development, can influence the TPC in the plants [22,24,35]. Thus, this explains the large differences observed between our and previous findings.

### 3.4. Antioxidant Capacities

The antioxidant capacities of the leaf and root extracts of the plants were investigated by various free radical scavenging assays, including DPPH, ABTS, and FRAP assays. In all of the antioxidant activity assays, the extract of leaves exhibited stronger antioxidant activity as compared to the extract of roots, which is in accordance with TPC and TFC.

**Figure 2:**
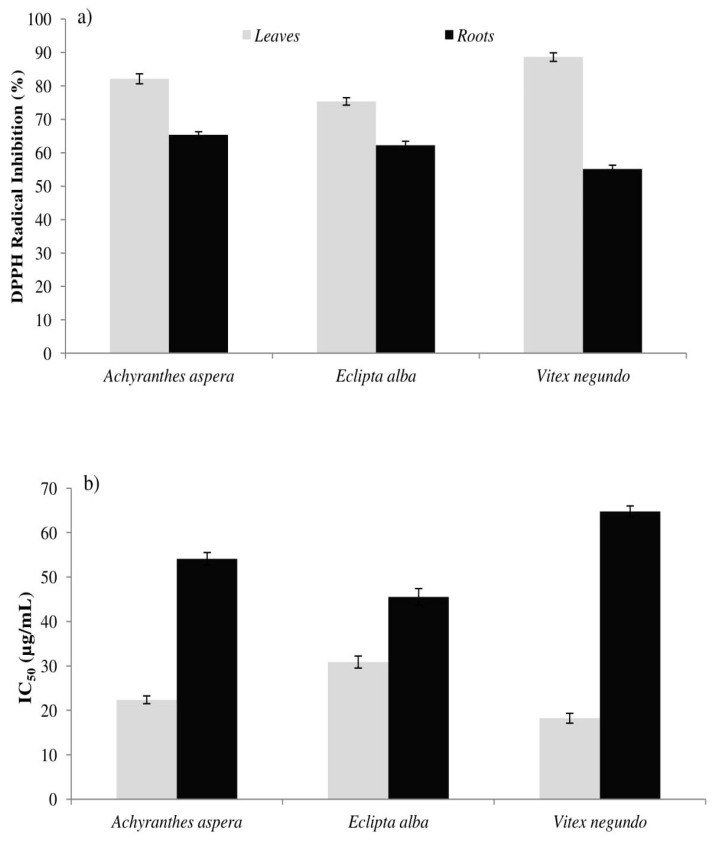
(%) inhibition of 2, 2-diphenyl-1-picrylhydrazyl radical (DPPH) free radical (**a**) and IC_50_ (**b**) value of the samples.

Figure 2 represents the % inhibition of the DPPH free radical (a) and IC_50_ value (b) of the leaf and root extracts. The highest DPPH inhibition (86.65% inhibition) was observed in the *Vitex negundo* leaf, while the root of the same species showed the lowest inhibition (55.15% inhibition). The leaf extracts showed more potent scavenging activity than their root counterparts (*p* < 0.001). Adedapo et al. [29] also observed a similar trend but only at higher concentrations of the extract. Antioxidant activity measured by DPPH assay in different vegetables available in the local market ranged from 0.61 to 8328.80 µmol trolox equivalent per gram [33]. At 100 µg/mL, Adedapo et al. [29] noticed an 89.7% and 67.0% inhibition of DPPH by the leaf and stem of a South African medicinal plant, respectively, while Shahat et al. [31] recorded almost 100% inhibition of DPPH by Asteraceae family plants. Dasgupta & De [7] reported IC_50_ values of some leafy vegetables in India, measured by DPPH assay, ranging from 61.4 to 1946  µg/mL, which are higher values than what we observed in this study.

Table 2 represents the antioxidant activity of the plant extracts (leaf and root) based on ABTS and FRAP assays. The existence of antioxidant substances or reductants in the plant extracts directs the conversion of the ferric (Fe^3+^) complex to the ferrous (Fe^2+^) form, which is the principle of FRAP assay. The decrease in Fe^3+^ in the solution leads to the decrease in color, which implies the potent reducing power of the plant extracts. Among the selected plant extracts, *Vitex negundo* leaf and root extracts showed the highest (554.41 ± 2.38) and lowest (53.78 ± 0.98) FRAP values, respectively (Table 2). 

In this study, the free radical scavenging power was also evaluated using the improved ABTS^•+^ method. The principle behind this assay is producing the ABTS radical cation (ABTS^•+^), a blue-green chromogen, by reacting ABTS and potassium persulphate. In the presence of antioxidant components, the color of the free radical is decreased, which has a characteristic absorbance at 734 nm [25]. Like DPPH inhibition and FRAP, *Vitex negundo* leaf and root extracts also showed the highest (282.41 ± 1.25) and lowest (7.50 ± 0.10) TEAC values, respectively (Table 2). Adedapo et al. [29] reported higher values of FRAP in stem than leaves, whereas in this study, we observed the opposite. However, a similar trend to our observation was reported by Adedapo et al. [29] in terms of ABTS value. The ABTS and FRAP values in some Indian leafy vegetables varied from 18.3 to 71.8 μmol trolox/g DW and 107.7 to 275.6 μmol Fe^2+^/g DW, respectively [28]. The antioxidant competencies obtained from FRAP assay and those obtained from TEAC assay were highly correlated (R = 0.988) (Table 3), which signifies that antioxidants present in these plants were proficient in scavenging free radicals (ABTS^•+^) and reducing oxidants (ferric ions).

Plants rich in secondary metabolites including phenolics and flavonoids demonstrate powerful antioxidant properties, both in vitro and in vivo, which is attributed to their redox properties and chemical structures [35,36,37]. The results of the previous studies are also similar to ours [28,30,31,32,33,38]. Several studies reported a strong correlation between antioxidant activity and TPC, indicating the importance of polyphenols as a potent antioxidant component, which is emerging as a trend in numerous plant varieties [28,30,31,32,33,38]. In this study, we also observed that the higher TPC of the plant extracts resulted in higher antioxidant activity; moreover, we found that the relationship between the antioxidant capacity and phenolic compounds of the extracts was positively correlated (Table 3), accordingly signifying that phenolic compounds are major contributors to the antioxidant activity of the selected plant samples. Antioxidant molecules can neutralize the reactive free radicals and prevent the progression of chronic diseases, including diabetes, cancers, cardiovascular diseases, neurodegeneration, and inflammatory mediated diseases. Antioxidant activity from foods is normally generated from the combination of several compounds rather than a specific single compound, and hence it is difficult to relate the antioxidant activity to a specific compound [31]. Therefore, a diet supplemented with different wild plants can supply different antioxidant molecules and subsequently provides preventive measures. Thus, these wild edible plants, as a source of rich antioxidant compounds, should be brought to the attention of the general population as important health-promoting foods. Since this is the first study on the antioxidant activity of the selected plants, a detailed phytochemical analysis is an absolute necessity to isolate the active phenolic and flavonoid components.

### 3.5. α-Amylase Inhibitory Activity of the Selected Plants

**Figure 3:**
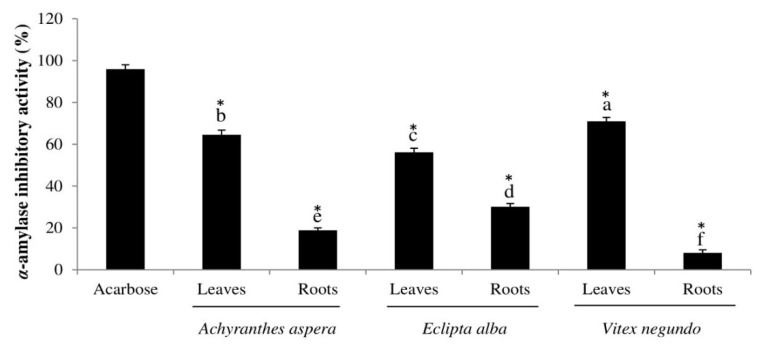
α-amylase inhibitory activity (%) of acarbose and the selected plant samples. Means that do not share a letter among samples are significantly different. * *p* < 0.05 compared to acarbose.

The potential of the plant extracts to inhibit α-amylase activity was analyzed (Figure 3). The result revealed that the *Vitex negundo* leaf extract showed the highest inhibitory activity against α-amylase (70.95% inhibition) whereas the leaf extracts of *Achyranthes aspera* and *Eclipta alba* inhibited α-amylase by 64.49% and 56.16%, respectively. The lowest inhibitory activity (8.05% inhibition) was observed in the root extract of *Vitex negundo*. These plant extracts, especially the leaf extracts, showed appreciable α-amylase inhibitory effects when compared with acarbose and their root counterparts (Figure 3). Olubomehin et al. [39] also found higher α-amylase inhibition by the leaf compared to root of a traditional Nigerian plant. From Table 3, it can also be seen that the phenolic compounds exhibited significant correlation in inhibiting α-amylase activity. This result is also comparable to previous study findings using other Bangladeshi [40], Indian [41], and Egyptian wild plants [26]. On the other hand, some authors reported no significant inhibition on α-amylase activity by traditional plants [42]. Restraining or limiting the activity of α-amylase is one of the approaches in the prevention and/or management of type-2 diabetes. The inhibition of α-amylase delays carbohydrate absorption after food ingestion and thereby decreases the rate of glucose production and eventually lowers blood glucose levels [19]. Therefore, the leaves of wild plants used in this study could be used as functional food ingredients for regulating and maintaining carbohydrate metabolism and postprandial hyperglycemia.

### 3.6. Principal Component Analysis

**Figure 4:**
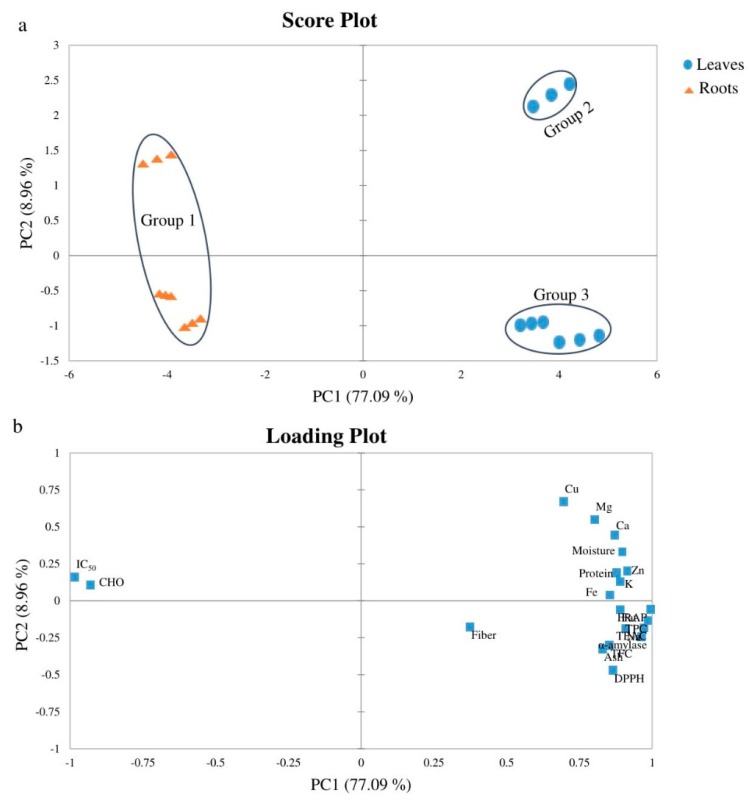
Score (**a**) and loading (**b**) plots of PCA analysis for the first and second components of selected plants.

PCA analysis of pooled proximate variables, antioxidant activities, total phenolic content, total flavonoid content and α-amylase activity of the selected wild plants was carried out. In the PCA analysis, the first two principal components explained about 86.05% of the total variance (Figure 4): PC1 (77.09%) and PC2 (8.96%). The IC_50_ and CHO were negatively associated with PC1, whereas the loadings on PC2 specified high contributions from DPPH and Cu, with negative and positive values, respectively.

Figure 4a,b represents the score and loading plots of analyzed variables of the plant samples, respectively. In Figure 4a, a clear separation between the leaves and roots of the analyzed sample was observed. Also, in Figure 4a, the formation of three groups can be seen. Group 1 consists of roots of *Vitex negundo*, *Achyranthes aspera* & *Eclipta alba*. Groups 2 & 3 consist of leaves of *Eclipta alba,* and *Achyranthes aspera* & *Vitex negundo*, respectively. IC_50_ and CHO variables contributed mostly to the separation of group 1, moisture, protein and all of the minerals except sodium for group 2, and TPC, TFC, α-amylase, antioxidant activities (DPPH, FRAP & TEAC), sodium, fat, protein, ash and fiber for group 3.

## 4. Conclusions

The results suggest that the leaves and roots of *Achyranthes aspera, Eclipta alba* and *Vitex negundo* are a promising source of fiber, protein, minerals, antioxidant molecules and could serve as material for dietary supplementation and functional food ingredients. Our investigation of the selected plants also provides in vitro evidence of α-amylase inhibition and justifies their use in the management of diabetes. However, the isolation of active compounds and the in vivo antidiabetic potential of these plants warrant further studies.

## Figures and Tables

**Table 1:** Proximate composition (g/100g dry weight (DW)), and macro minerals and micro minerals (DW basis) of selected wild plants.

Wild Plants	*Achyranthes aspera* L. (Upat Lengra)		*Eclipta alba* L. (Kalokeshi)		*Vitex negundo* L. (Nirgundi)	
	Leaves	Roots	Leaves	Roots	Leaves	Roots
Proximate composition (g/100 g sample)						
Moisture	83.71 ± 1.33	61.23 ± 1.01	88.13 ± 1.55	55.44 ± 2.22	82.78 ± 2.68	70.41 ± 2.11
Protein	18.13 ± 1.67	7.08 ± 0.33	21.56 ± 1.10	13.21 ± 0.93	19.27 ± 0.85	11.35 ± 1.05
Fat	1.88 ± 0.20	0.89 ± 0.07	2.17 ± 0.11	0.94 ± 0.05	3.13 ± 0.51	1.12 ± 0.10
Fiber	18.65 ± 1.23	22.26 ± 0.56	20.28 ± 0.92	16.56 ± 0.52	19.70 ± 0.90	13.78 ± 1.34
Ash	21.43 ± 0.33	13.42 ± 0.88	19.78 ± 0.42	17.37 ± 0.51	20.15 ± 0.75	8.21 ± 0.61
Carbohydrate (CHO)	39.91 ± 1.85	56.35 ± 1.46	36.21 ± 0.63	51.92 ± 1.50	37.75 ± 0.75	65.54 ± 1.78
Mineral Composition						
Macro minerals (mg/100 g sample)						
Sodium (Na)	497.51 ± 3.66	135.20 ± 1.03	345.33 ± 1.25	100.50 ± 0.70	577.82 ± 2.23	202.72 ± 1.08
Potassium (K)	4866.45 ± 5.78	1185.37 ± 1.75	5174.82 ± 5.74	2359.90 ± 4.01	3345.20 ± 4.65	1058.39 ± 5.05
Magnesium (Mg)	333.51 ± 2.43	164.38 ± 0.96	274.20 ± 3.98	148.21 ± 2.22	315.15 ± 2.45	190.80 ± 0.70
Calcium (Ca)	1493.45 ± 3.73	842.16 ± 2.02	2221.33 ± 6.83	523.91 ± 1.13	1786.24 ± 7.88	1090.90 ± 1.10
Micro minerals (mg/100 g sample)						
Iron (Fe)	31.61 ± 0.70	19.83 ± 1.33	45.22 ± 1.12	16.13 ± 0.80	62.05 ± 1.01	23.40 ± 0.9
Zinc (Zn)	6.03 ± 0.09	3.51 ± 0.05	5.82 ± 0.96	2.80 ± 0.09	5.88 ± 0.44	4.35 ± 0.65
Copper (Cu)	1.13 ± 0.02	0.51 ± 0.01	2.33 ± 0.07	0.67 ± 0.03	1.08 ± 0.04	0.84 ± 0.02

**Table 2:** Total phenolic, flavonoid, TEAC, and FRAP contents of the selected samples.

Scientific Name	Family	Local Name	TPC ^1^ (mg GAE/g DW)		TFC ^2^ (mg QE/g DW)		TEAC ^3^ (µmol trolox/g DW)		FRAP ^4^ (µmol Fe^2+^/g DW)	
			Leaves	Roots	Leaves	Roots	Leaves	Roots	Leaves	Roots
*Achyranthes aspera* L.	Amaranthaceae	Upat Lengra	68.84 ± 0.61^a^	4.55 ± 0.11^b^	80.23 ± 0.55^a^	2.23 ± 0.19^b^	250.18 ± 1.08^a^	12.13 ± 0.28^b^	505.19 ± 1.56^a^	65.22 ± 0.70^b^
*Eclipta alba* L.	Asteraceae	Kalokeshi	55.32 ± 0.47^b^	8.45 ± 0.15^a^	31.55 ± 0.25^c^	4.88 ± 0.31^a^	184.31 ± 1.42^b^	18.58 ± 0.20^a^	474.35 ± 1.88^b^	81.05 ± 0.55^a^
*Vitex negundo* L.	** Lamiaceae	Nirgundi	72.11 ± 0.73^a^	2.46 ± 0.06^c^	51.07 ± 0.88^b^	1.22 ± 0.09^b^	282.41 ± 1.25^a^	7.50 ± 0.10^b^	554.41 ± 2.38^a^	53.78 ± 0.98^c^

Values in the same column having different letters differ significantly (*p* < 0.05). ^1^ Total phenolic content; ^2^ Total flavonoid content; ^3^ Trolox equivalent antioxidant capacity; ^4^ Ferric reducing antioxidant power. FRAP: ferric-reducing antioxidant power; TEAC: trolox equivalent antioxidant capacity; TPC: total phenolic content: GAE: gallic acid equivalent; TFC: total flavonoid content; QE: quercetin equivalent.

**Table 3:** Pearson correlation and corresponding *p*-values among variables.

**Correlation Matrix**						
**Variables**	**TPC**	**TFC**	**TEAC**	**DPPH**	**FRAP**	**α-Amylase**
TPC		0.916	0.996	0.906	0.996	0.974
TFC	0.916		0.916	0.829	0.884	0.876
TEAC	0.996	0.916		0.914	0.988	0.966
DPPH	0.906	0.829	0.914		0.883	0.963
FRAP	0.996	0.884	0.988	0.883		0.967
α-amylase	0.974	0.876	0.966	0.963	0.967	
***p*-Values**						
**Variables**	**TPC**	**TFC**	**TEAC**	**DPPH**	**FRAP**	**α-Amylase**
TPC		<0.0001	<0.0001	<0.0001	<0.0001	<0.0001
TFC	<0.0001		<0.0001	<0.0001	<0.0001	<0.0001
TEAC	<0.0001	<0.0001		<0.0001	<0.0001	<0.0001
DPPH	<0.0001	<0.0001	<0.0001		<0.0001	<0.0001
FRAP	<0.0001	<0.0001	<0.0001	<0.0001		<0.0001
α-amylase	<0.0001	<0.0001	<0.0001	<0.0001	<0.0001	
